# Mutagenicity of the organic fraction of World Trade Center dust

**DOI:** 10.1002/em.22519

**Published:** 2022-12-13

**Authors:** David M. DeMarini, Sarah H. Warren, Lance R. Brooks

**Affiliations:** 1 Biomolecular and Computational Toxicology Division, Center for Computational Toxicology and Exposure, Office of Research and Development, U.S. Environmental Protection Agency, Research Triangle Park, North Carolina, USA; 2 Homeland Security and Materials Management Division, Center for Environmental Solutions and Emergency Response, Office of Research and Development, U.S. Environmental Protection Agency, Research Triangle Park, North Carolina, USA

**Keywords:** carcinogenicity, mutagenicity, *Salmonella*, SRM 1649b, World Trade Center dust (WTC)

## Abstract

Most studies of the health effects and chemical characterization of the dust resulting from the catastrophic collapse of the World Trade Center (WTC) on September 11, 2001, have focused on the large inorganic fraction of the dust; however, chemical analyses have identified mutagens and carcinogens in the smaller organic fraction. Here, we determined the mutagenicity of the organic fraction of WTC dust in *Salmonella*. Only 0.74% of the mass of the particulate matter (PM) <53 μm in diameter was extractable organic matter (EOM). Because the EOM was 10 times more mutagenic in TA100 +S9 than in TA98 +S9 and was negative in TA98 −S9, we inferred, respectively, that polycyclic aromatic hydrocarbons (PAHs) played a role in the mutagenicity and not nitroarenes. In TA98 +S9, the mutagenic potency of the EOM (0.1 revertant/μg EOM) was within the range of EOMs from air and combustion emissions. However, the EOM-based mutagenic potency of the particles (0.0007 revertants/μg PM) was 1–2 orders of magnitude lower than values from a review of 50 combustion emissions and various air samples. We calculated that 37 PAHs analyzed previously in WTC EOM were 5.4% of the EOM mass and 0.04% of the PM mass; some air contained 0.3 μg WTC EOM/m^3^ (0.02 μg PAHs/m^3^). Populations exposed to WTC dust have elevated levels of prostate and thyroid cancer but not lung cancer. Our data support earlier estimates that PAH-associated cancer risk among this population, for example, PAH-associated lung cancer, was unlikely to be significantly elevated relative to background PAH exposures.

## 1 ∣ INTRODUCTION

The collapse of the World Trade Center (WTC) on September 11, 2001, caused the immediate deaths of thousands of people and emitted an estimated 1 million tons of material into the surrounding environment (Offenberg et al., 2003) with immediate and likely long-term health and environmental consequences (Landrigan et al., 2004). The atmospheric plume was formed from pulverized cement, glass, and the contents of the buildings and contained calcium sulfate (gypsum), calcium carbonate (calcite), glass fibers, asbestos, metals, soot, volatile organic compounds (VOCs), and combustion products such as polycyclic aromatic hydrocarbons (PAHs) (Clark et al., 2003; Lioy et al., 2002; McGee et al., 2003).

People at the greatest risk of exposure were first responders, such as firefighters, police, and paramedics, as well as construction workers and volunteers who worked for months at the 16-acre site known as Ground Zero (CDC, 2002; Prezant et al., 2002). The composition of the dust particulates inside buildings was similar to that found outside, suggesting that residents also received indoor exposures to the same dust as that outside (Yiin et al., 2004). An estimated 100–1000 tons of PAHs may have been generated from the collapse of the buildings (Offenberg et al., 2003). However, only ~1 to ~2% of the mass of the settled dust was particulate matter <2.5 μm in diameter (PM_2.5_); most was >53 μm (PM_53_) (Lioy et al., 2002), leaving unresolved whether the population within the vicinity of the site was exposed to high concentrations of airborne PAHs (Pleil et al., 2004).

A review published 5 years after the event (Gavett, 2006) showed that short-term exposure to high concentrations of WTC dust increased respiratory effects such as persistent cough, bronchial hyperreactivity to aerosolized methacholine, newly developed asthma, and gastroesophageal reflux disease. In the intervening years, there has been increasing documentation of an array of adverse health effects resulting from WTC dust exposures, including inflammation of the lung and lymph nodes (sarcoidosis) (Webber et al., 2017), pulmonary fibrosis (Li et al., 2019), reduced lung function and increased airway hyperreactivity (Cleven et al., 2021), and long-term cardiovascular disease associated with hypertension, hypercholesterolemia, and diabetes (Cohen et al., 2019).

Increased incidences of thyroid and prostate cancers have been associated with exposure to WTC dust (Boffetta et al., 2022; Hashim et al., 2018; Li et al., 2022; Solan et al., 2013; Webber et al., 2021), and these increases are likely WTC-specific and not due to increased medical surveillance of the population (Gong et al., 2019; Sunil et al., 2019; Tuminello et al., 2019). Singh et al. (2018) estimated that during the 20-year period of 2012–2031, the incidence of thyroid and prostate cancers would increase among the population exposed to WTC dust relative to a demographically similar population.

Other studies have not found elevated overall mortality, including that due to cancer, among rescue and recovery workers and community members exposed to WTC dust (Boffetta et al., 2022; Jordan et al., 2018; Li et al., 2022). A subset of New York City firefighters has shown resistance to loss of lung function, perhaps due to a protective metabolome in these individuals (Crowley et al., 2019). In contrast to prostate and thyroid cancers, the incidences of lung, colorectal, and kidney cancers have been predicted to decrease among the WTC dust-exposed population relative to a control population during the 20-year period of 2012–2031 (Singh et al., 2018). A recent evaluation by the International Agency for Research on Cancer (IARC) of the carcinogenicity to humans of occupational exposure as a firefighter, which included WTC studies but was not specific to WTC, found that such an occupational exposure causes mesothelioma (with asbestos as a plausible causal agent) and bladder cancer (with PAHs as a plausible causal agent) (Demers et al., 2022).

Twenty-one years have passed since the collapse of the towers; however, no epidemiology studies have reported an increase in lung cancer among the exposed population (Sigel et al., 2022). The only experimental carcinogenesis study of WTC particles found that multiple intratracheal instillations of the particles in Sprague–Dawley rats did not cause lung cancer, although some rare hemangiomas and hemangiosarcomas were observed (Soffritti et al., 2013). Thus, exposure to WTC particles has been associated with some cancers (prostate and thyroid) but not with others (lung).

Although WTC dust particles have been extensively analyzed both physically and chemically (Clark et al., 2003; Lioy et al., 2002; McGee et al., 2003), the percentage of extractable organic matter (EOM) has never been reported. Most studies have indicated that the majority of the mass of the particles is composed of inorganic material such as asbestos, glass fibers, metals, etc., and that most of the observed health effects are due largely to these and other components of the inorganic fraction of the particles (Gavett et al., 2003; Landrigan et al., 2004). Nonetheless, analyses of organic extracts of the particles have identified the presence of a wide array of organics, including some mutagenic and carcinogenic PAHs (Pleil et al., 2004; Swartz et al., 2003), polychlorinated biphenyls (PCBs) and organochlorine pesticides (Offenberg et al., 2003, 2004), as well as polychlorinated furans and dioxins (Clark et al., 2003; Lioy et al., 2002; McGee et al., 2003).

Here, we determined the % EOM of WTC PM_53_ to clarify the proportion of the particles composed of extractable organics. Although the organic fraction has been shown to contain a wide variety of known mutagens and carcinogens, no study has evaluated the mutagenicity of the organic fraction. Thus, we have done so here using the *Salmonella* mutagenicity assay and compared the mutagenic potencies of the EOM and the particles to those of a standard urban dust sample (SRM 1649b) and other particles from a variety of air and combustion emissions.

## 2 ∣ MATERIALS AND METHODS

### 2.1 ∣ Samples

We purchased the Standard Reference Material (SRM) 1649b Urban Dust from the National Institute of Standards and Technology (NIST). These particles, which are also called particulate matter (PM), were collected from ambient air in Washington, DC, and they have been chemically characterized for a large number of compounds covering a wide variety of chemical classes (NIST, 2016). The particles range in size from ~0.2 to ~100 μm, with the median particle size being ~20 μm (NIST, 2016). SRM 1649b is a reformulation of the original SRM 1649a sample, which had been evaluated for mutagenicity by May et al. (1992) but is no longer available. SRM 1649b had not been evaluated for mutagenicity prior to our study.

The WTC dust sample tested was WTC3, which was a sieved sample (<53 μm) obtained as described (McGee et al., 2003) 0.3 miles southeast of the center point of Ground Zero. WTC dust has been characterized chemically for many types of organic compounds among various chemical classes (Gavett et al., 2003; McGee et al., 2003; Offenberg et al., 2003, 2004; Pleil et al., 2004; Swartz et al., 2003). Thus, the WTC dust sample studied here was <PM_53_.

### 2.2 ∣ Organic extractions

We extracted organic compounds from the particles via sonication of the particles in dichloromethane (DCM), as described (DeMarini et al., 2017). Briefly, we sonicated the particles in DCM for 20 min, filtered the extracts through two 1-μm Zefluor^®^ filters, evaporated the sample to 10 ml, and then filtered the extract first through a 0.2-μm and then through a 0.02-μm Anotop^®^ filter. We also prepared blanks consisting of just DCM. We determined the percentage of extractable organic matter (% EOM) gravimetrically as described (Mutlu et al., 2016). We solvent-exchanged the DCM extracts into dimethyl sulfoxide (DMSO) at a concentration of 2 mg EOM/ml DMSO for SRM 1649b and at 8.974 mg EOM/ml DMSO for WTC.

### 2.3 ∣ Mutagenicity assays

We evaluated the organic extracts in the plate-incorporation assay of the *Salmonella* mutagenicity assay (Maron & Ames, 1983) as described (DeMarini et al., 2017) in the presence and absence of metabolic activation (S9). In a preliminary experiment in TA98 +S9, we evaluated the WTC dust EOM concentrate (8974 mg EOM/ml DMSO) at 18, 44.9, 89.7, 179.5, 448.7, and 897.4 μg EOM/plate; and we evaluated the SRM 1649b concentrate (2 mg EOM/ml DMSO) at 10, 25, 50, 100, and 200 μg EOM/plate. This preliminary experiment had a high number of spontaneous (control) revertants (rev)/plate (96 rev/plate), which was outside our laboratory's historical control range. Thus, the data from this experiment were not used in the analysis. However, based on the results, we modified the dose range such that subsequent experiments in all strain/S9 combinations with WTC dust were performed at 44.9, 89.7, 179.5, and 448.7 μg EOM/plate, and the doses for SRM 1649b were 25, 50, 100, and 200 μg EOM/ml DMSO. The metabolic activation (S9 mix) was made from liver S9 prepared from Aroclor-induced Sprague–Dawley rats (Moltox, Boone, NC). We evaluated the extracts in the frameshift strain TA98 with and without S9 and in the base-substitution strain TA100 with S9. The EOMs were tested at 1 plate/dose in 2 experiments; however, due to limited samples, the WTC dust EOM was tested at 1 plate/dose only once in strain TA98 −S9.

The genotypes of the strains have been described (Porwollik et al., 2001), and the classes of compounds the strains detect have been noted by Mutlu et al. (2013). In the presence of S9, both strains can detect PAHs, and TA98 +S9 can also detect aromatic amines and heterocyclic amines; in the absence of S9, TA98 detects nitroarenes (nitro-PAHs). In general, PAHs induce primarily base-substitution mutations and are, therefore, more potent in TA100 than in TA98 (DeMarini et al., 1994).

We incubated the plates for 3 days at 37°C and then counted the mutant colonies (revertants, rev) with an automatic colony counter (ProtoCOL 3, Synbiosis, Frederick, MD). We included negative controls (DMSO at 100 μl/plate) and positive controls with each experiment at 3 plates/dose. The positive controls were 2-aminoanthracene at 0.5 μg/plate for strains with S9 and 2-nitrofluorene at 3 μg/plate for strains without S9. We tested the blanks in each experiment at 1 plate/dose at 5 doses/experiment, where the highest dose was 100 μl of DMSO per plate.

We combined the data from replicate experiments and performed linear regression analyses over the initial linear portions of the dose–response curves to determine the mutagenic potencies (rev/μg EOM) ± standard error (SE) using Prism (GraphPad, San Diego, CA). We defined the initial linear portion of the curve by the line with the highest *r*^2^ value relative to that produced by inclusion of the lower doses. We considered samples mutagenic if they reached or exceeded a two-fold increase in mutagenicity (rev/plate) relative to the DMSO control and produced a significant slope value (*p* ≤ .05) based on a trend test of the regression (Prism, GraphPad, San Diego, CA). We used two-tailed unpaired t-tests to compare the mutagenic potencies of the EOMs. We determined the mutagenic potencies of the particles (rev/μg particle) by multiplying the mutagenic potency of the EOM (rev/μg EOM), which was the slope of the linear regression, by the % EOM. We expressed the mutagenicity of air samples as rev/m^3^ by multiplying the mutagenic potency of the particles (rev/μg particle) by the mass of particles in the air (μg PM/m^3^).

## 3 ∣ RESULTS

### 3.1 ∣ Percent EOM

There was no detectable EOM from the blanks; the % EOM of SRM 1649b urban dust was 2.5% and was 0.74% for WTC dust. We used these values to calculate the preparation of the DMSO concentrate and for converting the rev/μg EOM to rev/μg particle.

### 3.2 ∣ Mutagenic potencies of the EOMs

**FIGURE 1:**
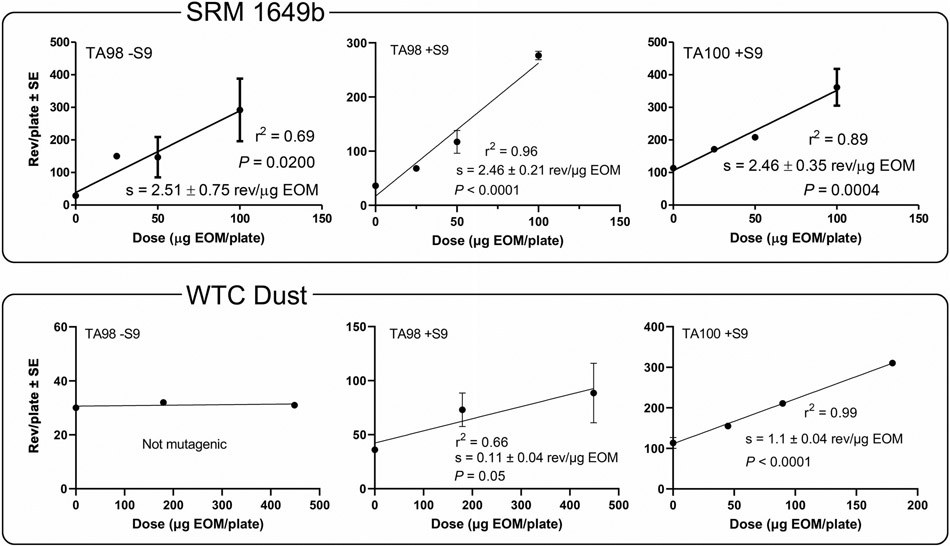
Mutagenicity dose–response curves of organic extracts of NIST SRM 1649b and WTC dust samples in the *Salmonella* plate-incorporation assay. Data are from Tables 1 and 2.

The primary mutagenicity data (rev/μg EOM) for the extracts are shown in Table 1 for SRM 1649b and in Table 2 for WTC dust; the blanks were not mutagenic, but the positive controls were. The mutagenicity data of the EOMs were used to construct the linear portions of the dose–response curves (Figure 1) from which the slopes were used to derive the EOM mutagenic potencies (Table 3).

The mutagenic potency of the EOM of SRM 1649b was identical among all three strain/S9 conditions (2.5 rev/μg EOM) (Table 3). Thus, for the EOM of SRM 1649b, the amount of mutagenic activity inferred to be due to PAHs (TA98 +S9 and TA100 +S9) was similar to the amount inferred to be due to nitroarenes (TA98 −S9). The EOM of WTC dust was not mutagenic in TA98 −S9 and, thus, exhibited no mutagenicity inferred to be due to nitroarenes. However, WTC dust EOM was 10 times more mutagenic in TA100 +S9 than in TA98 +S9 (*p* < .0001), indicating that it was more potent as a base-substitution mutagen than as a frameshift mutagen (Table 3). The requirement for S9 for the WTC EOM to be mutagenic in these strains and the greater potency in TA100 relative to TA98 suggest that PAHs likely accounted for much of this activity (DeMarini & Linak, 2022). The mutagenic potency of the EOM of SRM 1649b was 25 times greater than that of WTC dust in TA98 +S9, and it was twice as mutagenic as that of the WTC dust EOM in TA100 +S9 (*p* < .0001) (Table 3).

### 3.3 ∣ EOM-based mutagenic potencies of the particles

Multiplying the mutagenic potencies of the EOM (Table 3) by the % EOM gave the mutagenic potencies of the particles expressed as rev/μg particle (Table 4). For SRM 1649b, the mutagenic potencies of the particles were identical in all three strain/S9 conditions. The mutagenic potencies of the WTC particles were 10 times greater in TA100 +S9 than in TA98 +S9. The mutagenic potency of SRM 1649b particles was ~8 times greater than that of WTC particles in TA100 +S9. The mutagenic potency of SRM 1649b particles was 90 times greater than that of WTC particles in TA98 +S9 (Table 4).

## 4 ∣ DISCUSSION

### 4.1 ∣ Comparison of mutagenic potencies of EOM from various combustion emissions and air samples

Our finding that the % EOM of <PM_53_ WTC dust was only 0.74% confirms that the organic fraction comprised a miniscule portion of the mass of these particles and that 99.26% of the mass was inorganic material. For comparison, we attempted to extract organics from residual oil fly ash (ROFA) particles, which are also known to be largely inorganic in composition and cause a variety of cardiovascular effects in rodents (Dye et al., 1999; Farraj et al., 2009; Kodavanti et al., 1998). However, we were unable to obtain any EOM from ROFA particles (data not shown). The small percentage by mass of organics that composed WTC dust particles was among the lowest % EOM among 50 combustion emissions reviewed recently (DeMarini & Linak, 2022).

**FIGURE 2:**
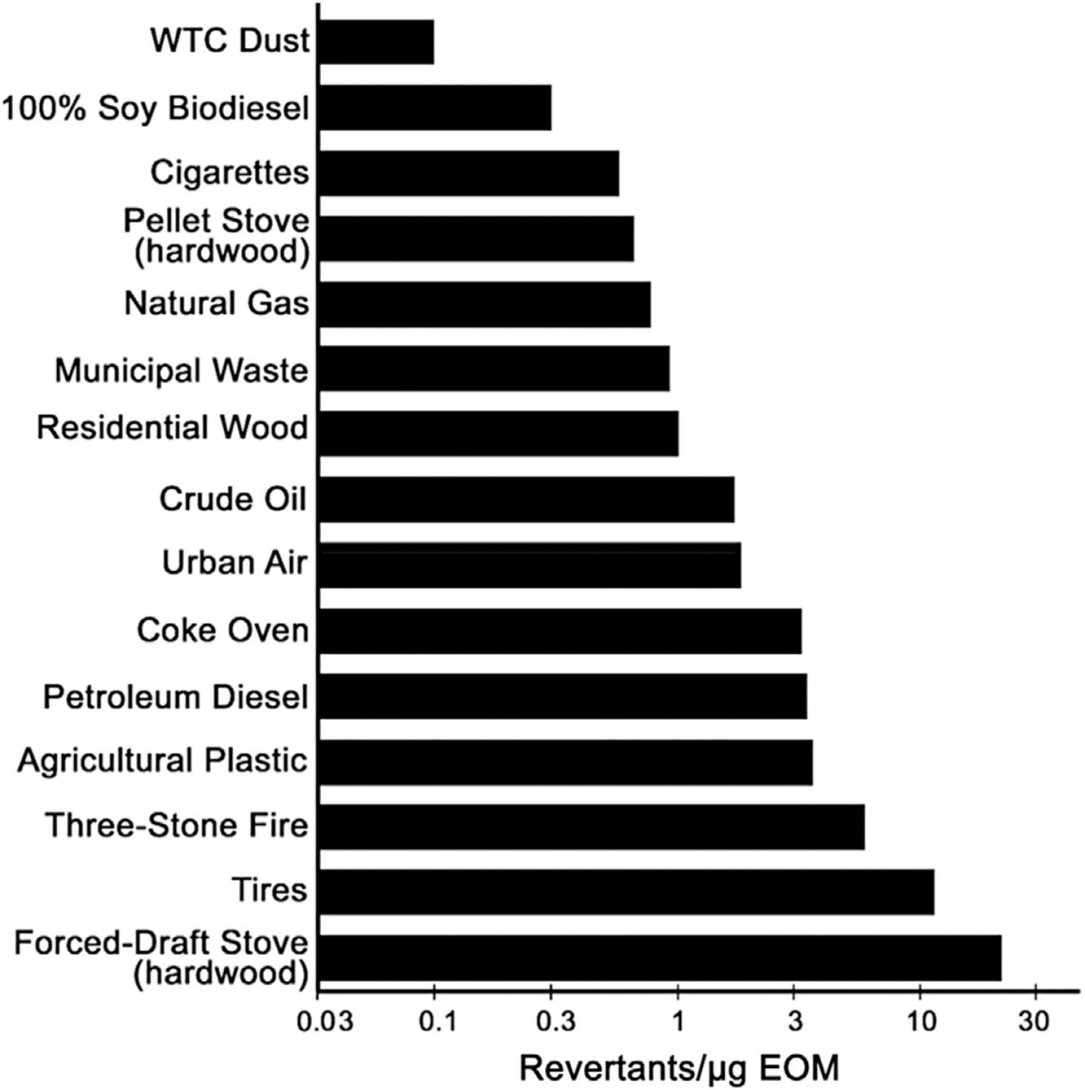
Mutagenic potencies of the extractable organic material (EOM) in *Salmonella* TA98 +S9 from a variety of combustion emissions. Figure from DeMarini and Linak (2022) with data for WTC Dust (top bar) added from Table 4. All EOMs were extracted using dichloromethane and were evaluated in the plate-incorporation assay in the lab of the authors.

The mutagenic potency of the EOM from the SRM 1649b particles was 2–25 times greater than that of WTC dust particles in TA100 +S9 and TA98 +S9, respectively (Table 3). Figure 2 compares the mutagenic potency of the EOM from WTC dust with that of the EOM from particles from various combustion emissions and air particles in strain TA98 +S9. The mutagenic potency of the EOM of WTC dust particles (0.1 rev/μg EOM) was 1–2 orders of magnitude lower than that of these particles (Figure 2). The mutagenic potency of the EOM of WTC dust particles is among the lowest of thousands of samples of air particles (IARC, 2016) and 50 combustion emissions (DeMarini & Linak, 2022), which together span ~2 orders of magnitude. Although on the low end, the mutagenic potency of WTC dust EOM is within the range of typical air and combustion emission PM.

In contrast, the mutagenic potencies of the WTC dust particles were ~8 to 90 times less than those of the urban dust standard SRM 1649b particles (Table 4). This is due to (a) the small percentage of mass of EOM per particle of WTC dust relative to SRM 1649b, (b) the weaker mutagenic potency of WTC EOM relative to that of SRM 1649b, and (c) possibly the size of the particles. WTC was <53 μm, and SRM 1649b was 0.2–100 μm, with a median particle size of 20 μm. Studies of atmospheric PM fractionated by size show that particles <2.0–3.3 μm in diameter are more mutagenic than larger particles, due to the condensation of organics onto the surface, with smaller particles having a larger surface-to-volume ratio than larger particles (Pagano et al., 1996). However, this is not always the case, as shown by de Kok et al. (2005), who found that for only two samples out of six taken from different locations in Maastricht, The Netherlands, the PM_2.5_ particles have mutagenic potencies in TA98 +S9 that were greater than those of PM_10_ or total suspended particulate (TSP) from the same location. Thus, the role of particle size on the % EOM and the mutagenic potency of the EOM is unclear.

### 4.2 ∣ Comparison of mutagenic potencies of various air particles

**FIGURE 3:**
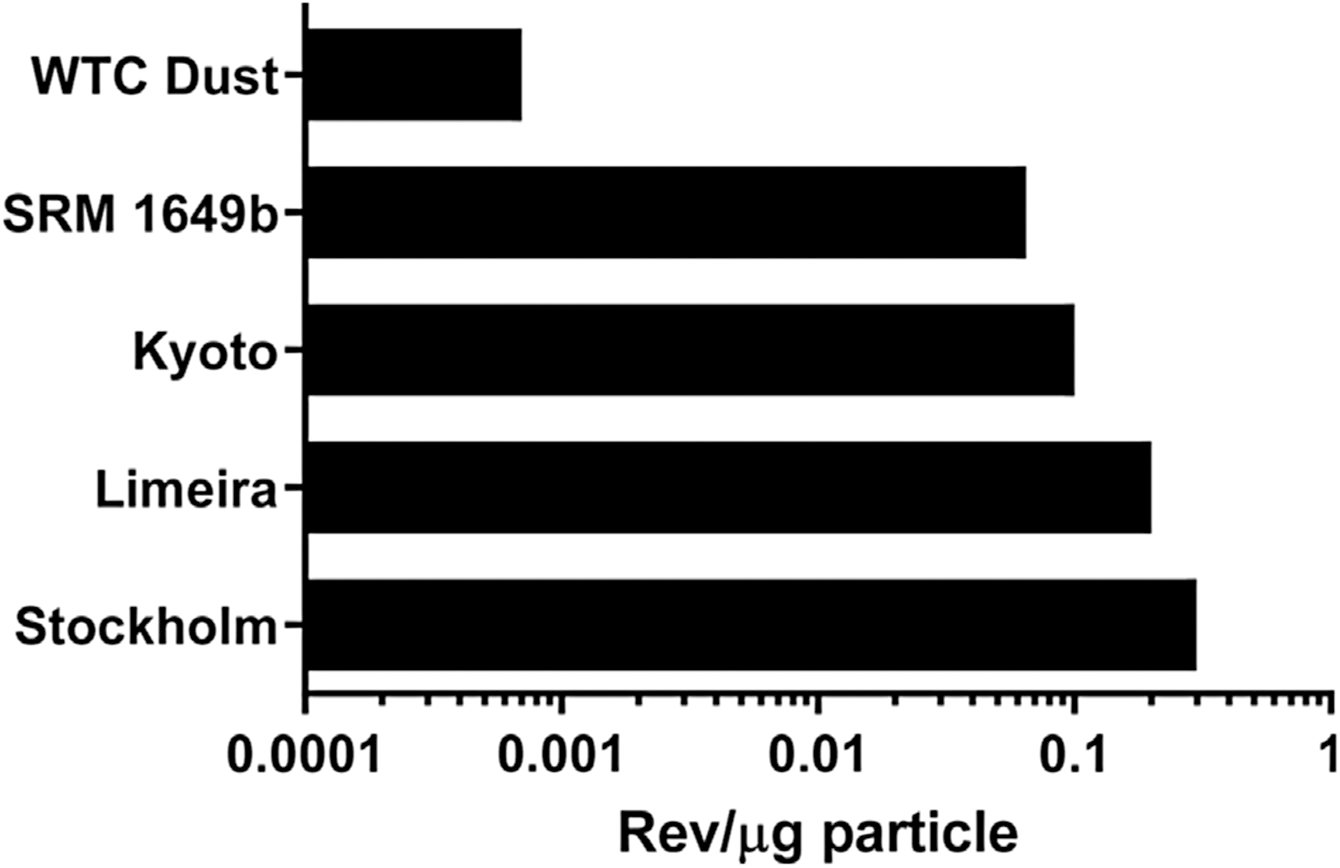
EOM-based mutagenic potencies in *Salmonella* TA98 +S9 of particles from various air and combustion emissions; data are from Table 5.

The mutagenic potency of WTC dust particles (rev/μg particles) was 90–429 times lower than that of SRM 1649b particles and ambient air particles from various cities (Stockholm, Kyoto, and Limeira) (Table 5, Figure 3), and it was 10 times lower than the lowest among the particles from 50 combustion emissions reviewed recently (DeMarini & Linak, 2022). As noted above, the SRM 1649b particles ranged from 0.2 to 100 μm, with a median size of 20 μm, and the WTC dust particles were <53 μm, resulting in an overlap in the size range between the two types of particles. The particles evaluated in the 3 cities were all total suspended particles (TSP), and the particles among the 50 combustion emissions reviewed by DeMarini and Linak (2022) were a variety of sizes, ranging from TSP to PM_2.5_. Thus, it is unclear to what extent particle size accounts for the different mutagenic potencies among these particles. However, for the other two factors that would influence the mutagenic potency of particles, namely the % EOM and the mutagenic potency of the EOM, the values for these two factors for WTC dust were among the lowest of typical cities and various combustion emissions.

### 4.3 ∣ Comparison of EOM-based mutagenic potencies of atmospheres

**FIGURE 4:**
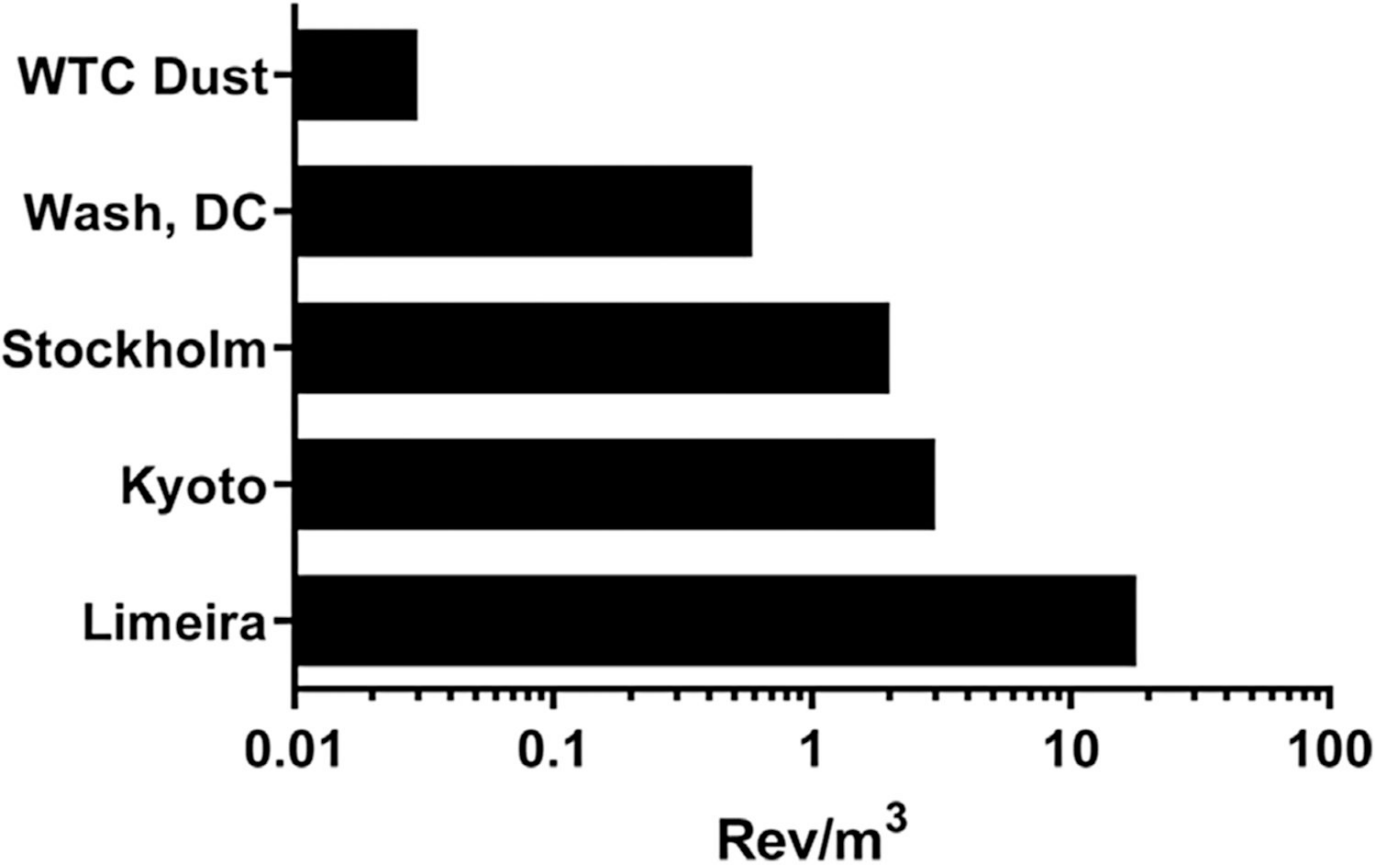
EOM-based mutagenic potencies in *Salmonella* TA98 +S9 of the air from various cities; data are from Table 5.

Multiplying the available data for μg of particles/m^3^ of air by the mutagenic potency of the particles (rev/μg particles) permits one to calculate the EOM-based mutagenic potency of the air (atmospheric mutagenic potency) expressed as rev/m^3^ of air. Such an analysis is shown in Table 5 and Figure 4 for the samples evaluated here as well as for air from other cities in strain TA98 +S9. The μg of PM_2.5_/m^3^ during the first month after the 9/11 event fluctuated from ~10 to 40 μg/m^3^, with a spike near September 26th of ~60–70 μg/m^3^ (Landrigan et al., 2004). Using the upper limit of the fluctuation (40 μg PM_2.5_/m^3^), the EOM-based atmospheric mutagenic potency during the month following the collapse of the buildings was 0.03 rev/m^3^ (Table 5). Using the 3-year average concentration of PM_2.5_ in Washington, DC, from 2016 to 2018 (U.S. EPA, 2018), the EOM-based atmospheric mutagenic potency was 0.57 rev/m^3^ (Table 5). Results from other cities are also shown in Table 5 and plotted in Figure 4.

Our analysis shows that the average EOM-based atmospheric mutagenic potency (rev/m^3^) of the <PM_53_ in the vicinity of Ground Zero for 30 days after the collapse of the buildings was 19 times less than that based on the SRM 1649b particles from Washington, DC (PM_20_); 67 times less than that of Stockholm, Sweden; 100 times less than that of Kyoto, Japan (PM_2.5_); and 600 times less than that of Limeira, Brazil (PM_2.5_), which is a city of ~300,000 people ~90 miles from Sao Paulo, Brazil (Table 5, Figure 4). These estimates underscore the limited contribution that the organic portion of WTC dust made to the mutagenicity of the air near Ground Zero during the month following the collapse of the buildings. Data from TSP collected from ambient air in cities in China and Japan show that the EOM-based atmospheric mutagenicity can exceed 5000 rev/m^3^ on days of high pollution (Coulibaly et al., 2016).

The most comprehensive analysis of the mutagenicity of outdoor air, based on >250 papers and >2500 air samples from around the world, found that there was generally less than a 10-fold variation (1 order of magnitude) in the mutagenic potency of organic extracts of PM, expressed as rev/μg EOM (IARC, 2016). Our data confirm this observation, with the 0.1 rev/μg EOM value of WTC dust being within ~1 order of magnitude of the other combustion emissions and air particles (Table 5). A recent study of major cities in Europe, Japan, and South America by Maselli et al. (2019) found that the EOM from all three cities had the same value of 2 rev/μg EOM (Table 5).

An analysis by IARC (2016) showed that although the mutagenic potency of the EOM of atmospheric PM does not vary more than an order of magnitude worldwide, the EOM-based atmospheric mutagenic potency expressed as rev/m^3^ varies by >5 orders of magnitude around the world. The estimated EOM-based atmospheric mutagenic potency of New York City air in the vicinity of Ground Zero during the 30 days following the collapse of the buildings (0.03 rev/m^3^) was 1–2 orders of magnitude lower than that of other major cities (Table 5, Figure 3). These observations highlight that the mutagenic potencies of the EOM from PM from widely different areas of the world, reflecting widely different sources of pollution, are rather similar in terms of mutagenic potency, suggesting that the organic chemical composition of such PM is also rather similar. However, the concentration of PM/m^3^ and, consequently, the EOM-based atmospheric mutagenic potencies of air sheds vary considerably (>10^5^) worldwide.

### 4.4 ∣ Lung cancer risk based on EOM exposure

**FIGURE 5:**
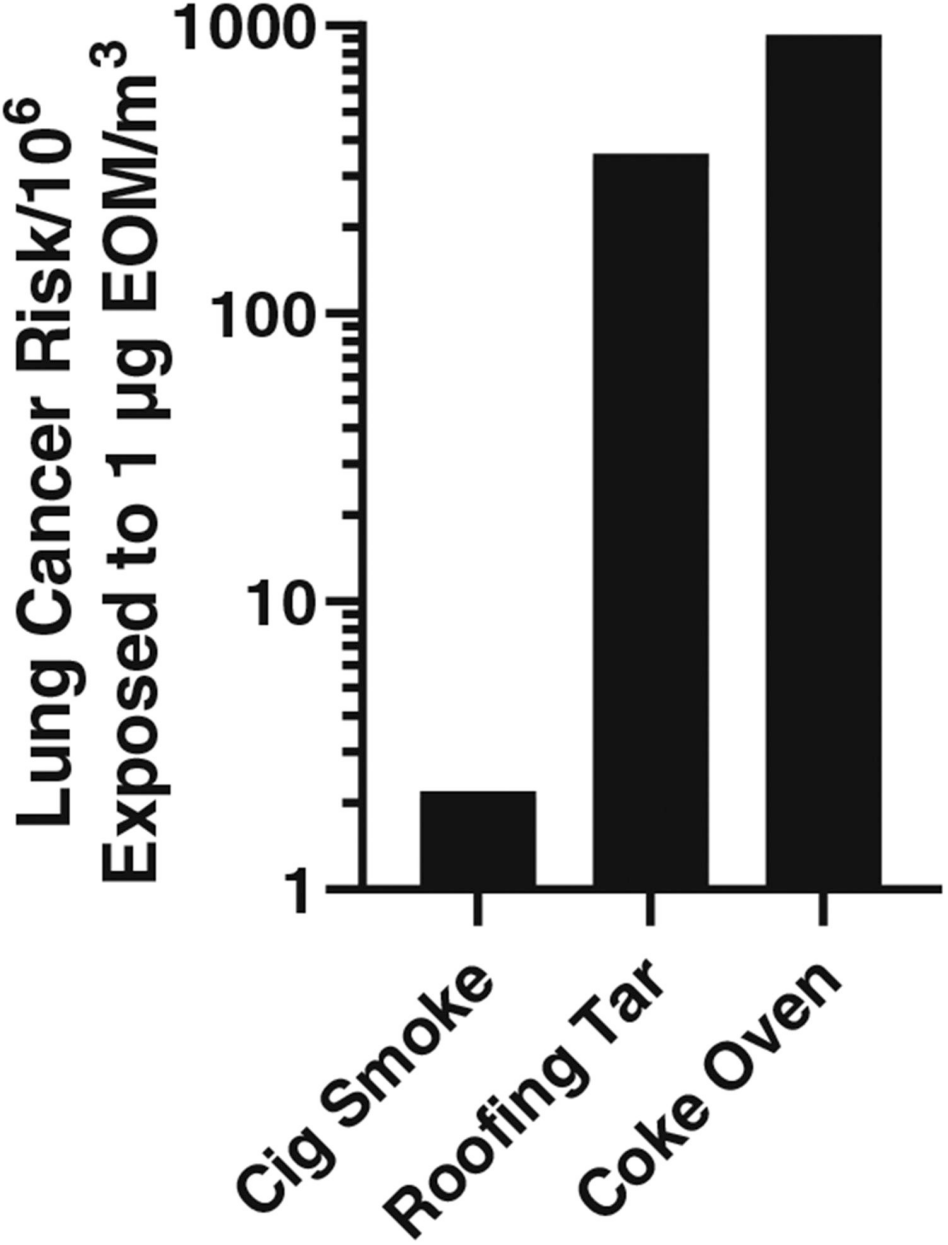
EOM-based risk for lung cancer; data from Albert et al. (1983). Cupitt et al. (1994) show the high correlation of these data with the carcinogenic potency of the EOMs on mouse skin, generating a standard curve from which an estimate of lung cancer risk can be made using the carcinogenic potency of EOMs on mouse skin. Such estimates have been made for air samples (Cupitt et al., 1994) and smoky coal emissions (DeMarini & Linak, 2022).

Exposure to mutagenic EOM is associated with increased cancer risk from a variety of complex mixtures and combustion emissions (Cupitt et al., 1994; DeMarini & Linak, 2022), including particles from outdoor air (IARC, 2016), coal emissions and woodsmoke (IARC, 2010), and diesel exhaust (IARC, 2014). As shown in Figure 5, the potential lung cancer risk resulting from a lifetime exposure to 1 μg EOM/m^3^ has been determined for the EOMs from various combustion emissions, including cigarette smoke, roofing tar, and coke oven emissions (Albert et al., 1983). Using the comparative potency bioassay method, Cupitt et al. (1994) found a nearly perfect correlation between the EOM-based lung cancer risk of Albert et al. (1983) (Figure 5) and the carcinogenic potency of the EOMs on mouse skin. They used the resulting linear curve to extrapolate and predict the EOM-associated lung cancer risk for various air samples for which the carcinogenic potency of the EOM had been determined on mouse skin, and we have done the same for the EOM from highly carcinogenic smoky coal emissions as shown in Figure 2 of DeMarini and Linak (2022).

Multiplying the upper value of the fluctuation of PM_2.5_ concentration during the 30 days following the collapse of the WTC buildings (Landrigan et al., 2004), which was 40 μg PM_2.5_/m^3^, by the % EOM for WTC dust particles (0.74%), indicates that such air contained 0.3 μg EOM/m^3^. If sufficient EOM from WTC dust could be obtained and tested in the mouse skin tumor assay, and the data were combined with our calculated concentration of 0.3 μg EOM/m^3^, the lung cancer risk for lifetime exposure to WTC dust could possibly be estimated.

### 4.5 ∣ Role of PAHs in the health effects of WTC dust

Although there is the possibility that PM_2.5_ WTC dust particles might be more mutagenic than <PM_53_ WTC dust particles, McGee et al. (2003) found that only 2.29–4.06% (~3%) of the mass of PM_53_ WTC dust was PM_2.5_. Offenberg et al. (2003) showed that the sum concentration of the 37 PAHs that they quantitated, which included the 16 EPA priority PAHs as well as 21 other PAHs that are analyzed routinely, accounted for only 0.04% of the mass of the PM_10-53_ and only 0.005% of the mass of the PM_2.5_.

As we have inferred, most of the mutagenic activity of the organic fraction of the PM_53_ was likely due to PAHs, all of which reside in the EOM, which accounted for only 0.74% of the mass of PM_53_ WTC dust. Thus, we estimate that the mass of the 37 PAHs accounted for 5.4% of the mass of the EOM. This was determined by assuming that if the mass of a PM_53_ particle was 1 g, then 0.74% (or 0.0074 g) of that mass was EOM. If 0.04% (or 0.0004 g) of the PM_53_ particle was the 37 PAHs, and all of that mass resided in the EOM, then 0.0004 g of PAHs out of 0.0074 g of EOM was 5.4%. Given that only 0.74% of the WTC PM was EOM and that 5.4% of the EOM was the 37 PAHs, then those 37 PAHs accounted for only 0.04% of the mass of the WTC <PM_53_, which is identical to the percentage determined by Offenberg et al. (2003) for WTC PM_10-53_. Based on the determination above that some WTC air had 0.3 μg EOM/m^3^ and that the mass of the 37 PAHs accounted for 5.4% of the mass of the EOM, we calculate (0.3 × 0.054) that such air had 0.02 μg PAHs/m^3^.

Among a wide array of combustion emissions, there is a Spearman *r* correlation of ~1 between the mutagenicity emission factor (revertant/megajoule_thermal_) and the PAH emission factor (μg PAH/megajoule_thermal_) (DeMarini & Linak, 2022). PAHs play a mechanistic role in the mutagenicity and carcinogenicity of PM in both rodents and humans, and the mutagenicity of the EOM from complex mixtures is a mechanistic component of the rodent and human carcinogenicity resulting from exposure to those mixtures (DeMarini & Linak, 2022; IARC, 2010, 2014, 2016).

Exposure to elevated levels of PAHs based on levels of PAH-DNA adducts in cord blood may have contributed to reduced fetal growth (Perera et al., 2005) and a modest reduction in cognitive development among children exposed prenatally to WTC dust (Perera et al., 2007). Relative to non-WTC-exposed firefighters, DNA sequence analysis of blood from WTC first responders showed increased clonal hematopoiesis, which is the acquisition of somatic mutations in blood cells and is associated with an increased risk of hematologic malignancies and inflammatory disorders (Jasra et al., 2022). Exposure of mice to WTC dust via oropharyngeal aspiration induced mutations in hematopoietic stem cells and progenitor cell compartments (Jasra et al., 2022). The small organic fraction of WTC dust may be a possible cause of these mutations; however, metals and chronic inflammation from the large inorganic fraction could also play a role. In general, the inorganic fraction of PM is not mutagenic; however, components of the inorganic fraction, such as some metals, are mutagenic and carcinogenic (IARC, 2012).

As discussed earlier, our data suggest that much of the mutagenic activity of the EOM from WTC dust was likely due to PAHs and not to nitroarenes. The only estimate of PAH-associated cancer risk among those exposed to WTC dust concluded that it was unlikely that such risk would have been elevated significantly above that due to background exposure to the average PAH concentrations to which residents of New York City would be exposed by living there for 70 years (Pleil et al., 2004). The authors estimated that there would be only a 10^−8^ increase in lifetime cancer risk among those at Ground Zero from WTC-associated PAHs based on the samples they analyzed. Our data showing the weak mutagenic activity of the organic (PAH-containing) fraction of WTC particles are consistent with this estimate.

Although there are no increases yet in PAH-associated cancers such as that of the lung among populations exposed to WTC dust (Sigel et al., 2022), there are well-documented increases in prostate and thyroid cancers among these populations (Boffetta et al., 2022; Hashim et al., 2018; Li et al., 2022; Solan et al., 2013; Webber et al., 2021). Prostate cancer has been associated with exposure to cadmium (IARC, 2012), which has been found in the inorganic fraction of WTC dust particles (Lioy et al., 2002). Thyroid cancer has been associated with iodine-131 and ionizing radiation (Krewski et al., 2019); however, it is unclear whether such agents were present in WTC particles or the Ground Zero environment. Although our data suggest that PAH-associated cancer risk among those exposed to WTC dust is unlikely to be elevated significantly relative to background PAH exposures, the role of the large inorganic and small organic fractions of WTC particles in the observed increased risk for prostate and thyroid cancers among populations exposed to WTC dust remains to be determined.

## Figures and Tables

**TABLE 1:** Mutagenicity (rev/plate) of EOM from SRM 1649b in *Salmonella*^a^

	TA98 +S9^b^		TA98 −S9		TA100 +S9	
Dose (μg EOM/plate)	Exp 1	Exp 2	Exp 1	Exp 2	Exp 1	Exp 2
0	35	37	30	28	123	104
25	64	72		150	179	164
50	132	102	85	209	208	208
100	282	271	196	388	418	305
200			316^c^			
Blank (100 μl/plate)	37	25	30	25	115	116
2-AA (0.5 μg/plate)^d^	489	528			262	635
2-NF (3 μg/plate)^e^			251	489		

a Data are counts from single plates, except for the DMSO controls (0 dose) and the positive controls, which are the average of 3 plates.

b As described in the Materials and Methods, a preliminary experiment in TA98 +S9 had an average spontaneous (0 dose) value of 96 rev/plate, which was outside of our laboratory control range; thus, this experiment was not used in the analysis and is not shown here.

c Not used to generate the regression in Figure 1 because this data point was outside of the initial linear portion of the dose–response curve as evidenced by *r*^2^-value.

d 2-AA is 2-aminoanthracene.

e 2-NF is 2-nitrofluorene.

**TABLE 2:** Mutagenicity (rev/plate) of EOM from World Trade Center dust in Salmonella^a^

	TA98 +S9^b^		TA98 −S9 Exp 1	TA100 +S9	
Dose (μg EOM/plate)	Exp 1	Exp 2		Exp 1	Exp 2
0.0	35	37	30	123	104
44.9					155
89.7					211
179.5	62	84	32	309	312
448.7	69	108	31	320^c^	
Blank (100 μl/plate)	31	35	33	112	101
2-AA (0.5 μg/plate)^d^	489	528		262	635
2-NF (3 μg/plate)^e^			251		

a Data are counts from single plates, except for the DMSO controls (0 dose) and the positive controls, which are the average of 3 plates.

b As described in the Materials and Methods, a preliminary experiment in TA98 +S9 had an average spontaneous (0 dose) value of 96 rev/plate, which was outside of our laboratory control range; thus, this experiment was not used in the analysis and is not shown here.

c Not used to generate the regression in Figure 1 because this data point was outside of the initial linear portion of the dose–response curve as evidenced by *r*^2^-value.

d 2-AA is 2-aminoanthracene.

e 2-NF is 2-nitrofluorene.

**TABLE 3:** Mutagenic potencies (rev/μg ± SE) of EOMs^a^

Sample	TA98 +S9	TA98 −S9	TA100 +S9
SRM 1649b	2.5 ± 0.2	2.5 ± 0.6	2.5 ± 0.4
WTC dust	0.1 ± 0.04	0.0	1.1 ± 0.04

a Data are the slopes of the regressions calculated from the data in Tables 1 and 2 and plotted in Figure 1. The mutagenic potency of the EOM of SRM 1649b was not significantly different among the strain/S9 conditions (*p* > .75 for all comparisons). However, the mutagenic potency of WTC dust was significantly different between TA98 +S9 and TA100 +S9 (*p* < .0001). For each strain, the mutagenic potencies of the EOMs of SRM 1649b were significantly different from those of WTC dust (*p* < .0001).

**TABLE 4:** EOM-based mutagenic potencies (rev/μg) of particles^a^

Sample	TA98 +S9	TA98 −S9	TA100 +S9
SRM 1649b	0.063	0.063	0.063
WTC dust	0.0007	0.0	0.008

a Data were calculated by multiplying the data in Table 3 by the % EOM, which was 2.5% for SRM 1649b and 0.74% for WTC dust.

**TABLE 5:** EOM-based mutagenic potencies of air samples in *Salmonella* TA98 +S9

Air sample	% EOM	Rev/μg EOM	Rev/μg particle	Rev/m^3^
WTC dust (NYC)^a^	0.74	0.1	0.0007	0.03
SRM 1649b (Wash, DC)^b^	2.5	2.5	0.063	0.57
Stockholm, Sweden^c^	15.0	2.0	0.3	2.0
Kyoto, Japan^c^	5.0	2.0	0.1	3.0
Limeira, SP, Brazil^c^	9.0	2.0	0.2	18.0

a Rev/m^3^ calculated by multiplying rev/μg particle by 40 μg PM_2.5_/m^3^, which was the upper value of the fluctuation of PM_2.5_ concentration during the 30 days following the collapse of the buildings, obtained from Figure 3 of Landrigan et al. (2004).

b Rev/m^3^ calculated by multiplying the rev/μg particle by 9.0 μg PM_2.5_/m^3^, which is the 3-year average (2016–2018) of PM_2.5_ concentration for Washington, DC (U.S. EPA, 2018).

c Data from Maselli et al. (2019); all three cities were evaluated using a toluene extract of total suspended particles (TSP) using the Kado et al. (1983) protocol.
